# Dosage Parameters in Pediatric Outcome Studies Reported in 9 Peer-Reviewed Occupational Therapy Journals from 2008 to 2014: A Content Analysis

**DOI:** 10.1155/2016/3580789

**Published:** 2016-02-02

**Authors:** Bryan M. Gee, Kimberly Lloyd, Nancy Devine, Erin Tyrrell, Trisha Evans, Rebekah Hill, Stacee Dineen, Kristin Magalogo

**Affiliations:** ^1^Department of Physical and Occupational Therapy, Idaho State University, Campus Mail Box 8045, Pocatello, ID 83201-8045, USA; ^2^Idaho State University, Pocatello, ID 83201-8045, USA

## Abstract

Occupational therapists determine the dosage when establishing the plan of care for their pediatric clients. A content analysis was conducted using 123 pediatric occupational therapy outcomes studies from 9 scholarly international occupational therapy journals. The parameters of dosage were calculated using descriptive statistics in order to obtain a representation of dosage available within the current collage of pediatric occupational therapy outcomes studies. The results revealed that most studies reported portions of dosage parameters within the published studies. The average findings for the subcomponents related to dosage were session length (minutes) *M* = 58.7, duration of plan of care (weeks) *M* = 12.1, session frequency (per week) *M* = 3.4, and total hours of therapy (hours) *M* = 18.1. This first attempt at describing and calculating dosage related to pediatric occupational therapy practice indicates that evidence is lacking within the published literature to adequately guide OT dosage decisions. Further research related to dosage in pediatric occupational therapy practice is needed.

## 1. Introduction

Occupational therapists (OTs) determine the intervention dosage when establishing the plan of care for their pediatric clients as a part of the routine occupational therapy process [1]. The dosage selected for the provision of occupational therapy services is likely to influence the overall effectiveness of the intervention provided [2]. Published research literature may provide evidence to guide the choices of OTs when making dosage decisions in order to achieve the best possible outcomes for the most reasonable cost. However, it is not currently known how prevalent published research is that includes data regarding parameters and decision making towards intervention dosage.

Dosage may be defined as the combination of the frequency of treatment sessions, intensity of the intervention provided, duration of the episode of care, and the type of intervention or interventions applied [2–4]. The combination of these parameters of dosage likely interact to produce the outcome achieved. Research addressing different combinations of frequency, intensity, and duration for a particular intervention or combination of interventions would provide valuable evidence to guide the choices of OTs when making dosage decisions in pediatric clinical practice.

There are multiple influences that could impact an OTs choice of dosage when establishing the plan of care for a pediatric client. In addition to the evidence available within the research literature, an OTs choice of dosage could be influenced by client factors (medical diagnosis, age, client/family/care provider goals, etc.) [3], practice setting dynamics (setting type such as school or outpatient, scheduling logistics, colleagues' choices) [5, 6] reimbursement (restrictions on the number of visits funded by insurance) [7], and the choice of interventions. Research is needed to identify and better understand the process OTs use to select the dosage for a client's plan of care and which factors influence dosage decisions the most.

A search of PubMed, Ovid, and CINAHL of the published literature for the previous 15 years revealed only one published article describing decision making of dosage for outcomes following provision of physical therapy and occupational therapy services for adult clients [8]. No literature was found describing the relationship between dosage and therapeutic outcome for physical therapy or occupational therapy interventions for the decision making for pediatric clients. Within the published literature, there is inconsistent reporting of frequency, duration, and intensity of services provided, with an apparent emphasis on the type of intervention used. The lack of evidence to support dosage decisions requires the reader to infer potential influences of different components of dosage, often with incomplete information. Research is needed to fill the gap in the literature regarding the components and parameters of dosage decisions in occupational therapy research and practice and how they influence the provision of service for pediatric populations.

The purpose of this study was to ascertain the dosage parameters included in published outcome studies related to pediatric occupational therapy practice in key occupational therapy journals between the years 2008 and 2014. The guiding research question for this study was the following: How frequently are dosage parameters published in pediatric OT outcome studies? Our hypotheses were as follows: (1) Dosage parameters will be included within published OT outcome studies addressing pediatric interventions and (2) the current literature will include more outcome studies with partial than complete dosage parameters. A focused content analysis of recently published research literature identified the breadth and depth of the evidence available to inform an OTs dosage decisions for pediatric clients. The information provided by this study may indicate how available evidence is for guiding dosage decisions in pediatric OT clinical practice and influence future research on the inclusion and investigation of dosage parameters. 

## 2. Methods

### 2.1. Operational Definitions

For consistency throughout the process of this content analysis, intervention was defined as “to assist the client in reaching a state of physical, mental, and social well-being; to identify and realize aspirations; to satisfy needs; and to change or cope with the environment” (AOTA, p. 652). Pediatric occupational therapy was defined as a scope of occupational therapy practice for children and youth (0–21 years old) who exhibit occupational performance deficits in the areas of work, leisure/play, social participation, activities of daily living/instrumental activities of daily living, and education [9, 10]. Intervention frequency was defined as the number of treatment sessions (AOTA, 2006) and intervention duration was the length of the intervention plan [11–13]. Intervention intensity referred to the length of each intervention session (i.e., 60 minutes) and the type of service delivery (i.e., 1 : 1 or group) [12, 14–17]. Using a format of categorization and analysis exemplified by May-Benson and Koomar, [7] dosage was defined as including aspects related to an intervention's duration (weeks), sessions per week, minutes per session, treatment session length, and total therapy hours. Each category of dosage, if provided, was used as a metric of analysis.

### 2.2. Inclusion Criteria

For this study published articles that reported quantitative, measurable outcomes for pediatric clients between birth to 21 years of age were included in the initial review. In addition, articles had to report on occupational therapy interventions that fell within the Occupational Therapy Practice Framework: 3rd Edition (OTPF) and targeted pediatric clients, their teachers, caregivers, and/or parents. Furthermore, to be included in the review, articles must have stated at least one or more of the parameters regarding the frequency, intensity, and/or duration of the intervention tested as defined previously. Articles published between 2008 and 2014 in one of the following occupational therapy specific journals were considered for this study (see Table 1).

### 2.3. Exclusion Criteria

For the purposes of this review, systematic reviews or qualitative studies were not included. In addition, articles published in nonoccupational therapy journals were excluded since they typically do not report their connection to the OTPF which may not focus on the interventions most relevant to occupational therapy. Articles were excluded if they did not report at least one of the parameters of frequency, intensity, or duration of therapy or if they measured nonintervention aspects of the occupational therapy process (i.e., evaluation tools).

### 2.4. Procedures

A total of 123 outcome studies ultimately were included within the analysis and are listed in the Appendix. Two researchers and five graduate research assistants were assigned to screen the most current seven years (2008–2014) of each occupational therapy journal online via the journal's website. We read the abstract and methods section of each article in each journal reviewed (see Table 1) to determine if the article met all of the inclusion criteria. In the event that access to an article was unavailable through the Idaho State University library or other web-based resources, only the abstract was reviewed to determine if the inclusion and exclusion criteria were likely to be met and an interlibrary loan request was needed.

After all of the targeted journals were screened, the first review of each article was conducted and the following information was recorded: duration (weeks) of services, number of sessions per week, duration of each session (minutes), total therapy hours, type of delivery (1 : 1 or group), sample size, and level of evidence. A second reviewer analyzed the same information from each article and tracked the number of disagreements. Any disagreements were then discussed by the first and second reviewer. If the first and second reviewers were unable to come to an agreement, the issues were brought to the weekly meetings for all reviewers to discuss and resolve. Conflicts and final decisions were documented for each article.

## 3. Results

**Figure 1:**
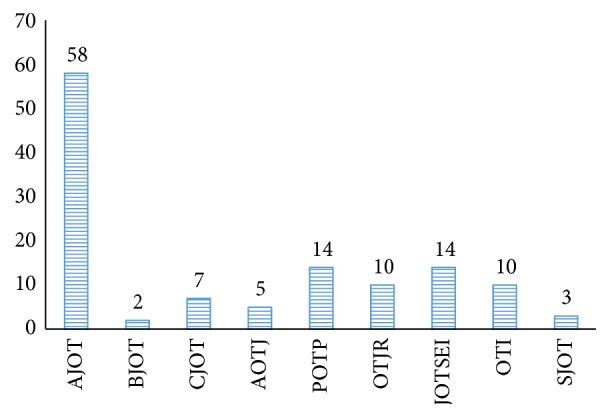
Frequency of articles from the journals reviewed. American Journal of Occupational Therapy: AJOT, British Journal of Occupational Therapy: BJOT, Canadian Journal of Occupational Therapy: CJOT, Scandinavian Journal of Occupational Therapy: SJOT, Physical and Occupational Therapy in Pediatrics: POTP, Occupational Therapy International: OTI, Journal of Occupational Therapy in Schools and Early Intervention: JOTSEI, Journal of Australian Occupational Therapy: AOTJ, and Occupational Therapy Journal of Research: OTJR.

One hundred and twenty three articles were found meeting the inclusion criteria. The data gathered from the articles representing sample size and the dosage parameters were analyzed using descriptive statistics (mean, median, and standard deviation) (see Table 2). Overall the American Journal of Occupational Therapy yielded the most pediatric outcomes studies (*n* = 58) with the remaining eight journals containing 14 or fewer during the previous seven years (see Figure 1).

All of the articles that met the inclusion criteria reported the number of participants who participated in each study (see Table 2) and there was a large range of sample sizes represented. The reporting of other subcomponents ranged from 75 to 88% (percentage equals the total *n* of articles divided by the *n* of articles reporting specific dosage subcomponents) of the articles. The length of each treatment session was the most reported subcomponent and the total number of therapy hours received was the least frequently reported (see Table 2).

## 4. Discussion

The purpose of this content analysis of pediatric outcome studies focusing on dosage was to investigate the availability of evidence to guide OTs making decisions for intervention planning with pediatric clients. The results of this study indicate that the majority of published outcome studies in pediatric OT journals that include at least one dosage parameter also include an additional dosage parameter. However, not all of the parameters are available within every article. This finding suggests there is some evidence available to clinically practicing pediatric OTs regarding some of the parameters of dosage needed when developing the plan of care. It also suggests that OTs currently must rely on clinical experience when selecting dosage parameters that are not fully represented within the literature. Further research and more complete documentation of dosage parameters are needed to ensure that the results of outcome studies may be applied clinically in an effective manner.

The results of this study display a wide range of OT practice and research that suggests a high intensity and frequency of intervention over shorter periods of time is used in outcome studies of pediatric occupational therapy interventions. It is not known whether the dosage of these interventions applied in clinical practice is consistent with the dosage used within the outcome studies. Our findings provide a clear depiction of how intervention dosage is represented when the aggregate of a large cluster of outcome studies are compiled for a single client population within one profession.

### 4.1. Duration

The duration of the plan of care was relatively frequently reported (86%) by the articles in this content review. Due to the heterogeneity of studies included in this review, the range of duration is substantial, spanning from 0.14 weeks to 96 weeks. The studies that were at the bottom and top end of the range and standard deviation for the duration of the length of plan of care in this content review may be heavily influenced by studies we labeled as “outliers.” For example, one study by Bates [18] was a case report of a single child who received outpatient occupational therapy services for almost two years. Ryan et al. [19], on the other end of the reported range, reported a total length of intervention of 1 hour (0.14 weeks) provided in a single session. The diversity of the duration of the plan of care reflected in our sample indicates the published literature does not yet provide a clear guideline for this dosage subcomponent.

### 4.2. Sessions per Week

Of the 98 studies that reported the frequency of sessions per week, 31 studies indicated that their intervention had occurred a single time per week. Conversely, on the other end of the reported range was a study by Lin, Lee, Chang, and Hong (2014) who explored the effectiveness of weighted vests with children diagnosed with ADHD and impulse control which reported 10 sessions per week of their intervention. The purpose and content of each study may have a strong influence on the number of sessions scheduled each week. The client's diagnosis and specific needs may require multiple, frequent sessions with interventions provided directly by a licensed therapist while other clients may benefit most from receiving less frequent care and having their home exercise plan progressed as appropriate. Clinically practicing pediatric OTs may use the results of this study to compare their own choices for selecting the frequency of sessions. However, the existing literature does not provide a clear guideline for selecting the frequency of sessions when developing the plan of care.

### 4.3. Minutes per Session

Similar to the length of the plan of care, the study at the top and the bottom of the overall range and size of the standard deviation is due to studies we labeled as “outliers.” An example may be found in Pizur-Barnekow et al. [20] who explored visual behavior and vagal reaction during structured activities of object perception with infants in a single intervention session of 4.5 minutes of treatment/session. Conversely, on the other end of the continuum de Brito Brandão et al. [21] explored the effectiveness of constraint therapy and bimanual training in children with cerebral palsy with their intervention lasting a duration of 360 minutes of treatment/day. These findings display the complexity inherent in making dosage decisions in OT pediatric practice and strongly support the need for further research to identify how the selection of the number of minutes per OT session is influenced by client factors such as age and diagnosis as well as the choice of intervention.

### 4.4. Total Therapy Hours

Similar to the length of the session, the studies with the longest and the shortest session length and size of the standard deviation we labeled as “outliers.” For example, Classen et al. [22] explored driver simulation for adolescents with ADHD and ASD that lasted a total of 20 minute or 0.33 of an hour. Conversely on the other end of the continuum Nwora and Gee [23] explored the effectiveness of a sound based intervention for children with PDD-NOS lasting a duration of 30 min/session and totaled approximately 1800 hours.

### 4.5. Type of Delivery

All 123 of the articles reviewed reported the type of intervention delivery (group, individual, or both) used. Sixty-two of the studies reported using an individual mode of therapeutic delivery, 49 used group-based intervention delivery, and four outcome studies used both types of delivery.

Our analysis also included a comparison of the types of research and the level of evidence sourced in Portney and Watkins [24] (see Table 3, levels of evidence). The most common type of research design found within our review was Level IV (case series, single case) (*n* = 51) and the less frequently found was Level I (randomized control trials).

### 4.6. Current Discussions of Dosage

Novak [25] stated that the core goal of evidenced based practice was for the therapist to “do the right things for the right client at the right time” (p. 1) in order to optimize therapeutic outcomes. Over the past several years the topic of therapeutic dosage has been brought up within the pediatric rehabilitation literature. A paucity of content related to dosage recommendations continues to plague pediatric practitioners. That being said, recent authors have attempted to address the topic of dosage through opinion pieces, the development of clinical pathways based upon medical/behavioral condition, or tiered decision scaffolds based upon the client's response to a given intervention. Palisono and Murr [26] argued for an approach where dosage was designed around the client's therapy readiness, skill level, and response to the intervention. Gannotti et al. [3] developed a clinical pathway as a method of approaching dosage (sessions per week, duration of session, and the type of specific motor/biomechanical intervention) for children with cerebral palsy (CP). The clinical pathway developed through targeting motor based interventions for children with CP is also uniquely infused with the core constructs of the International Classification System [27]. Bailes et al. [28] developed a clinical scaffold to serve as a guide for clinicians providing services which then informs them on some aspects of dosage (frequency) related to the client's response to therapy. The two clinical guides proposed by Gannotti et al. [3] and Bailes et al. [28] have yet to be explored further by way of clinician and client perspectives or intervention efficiency (greater outcomes in a shorter amount of time). Therefore, dosage is a current topic within the literature that warrants further research.

### 4.7. Implications for Clinical Practice

Our findings provide an opportunity for pediatric OTs to compare their current dosage decisions but do not provide the premise for demonstrating the most effective dosage parameters to select. In order for occupational therapy practitioners to incorporate evidence-based practice, to be adherent to the AOTA Code of Ethics (AOTA, 2010) and the 2017 American Occupational Therapy Association's Centennial Vision (Clark, 2010) [29, 30], a better understanding of current dosage decisions by OTs for pediatric interventions is needed. Exploring how dosage is being implemented in clinical research may assist the occupational therapy profession to establish evidence for the prescription of services as related to frequency, intensity, and duration to ensure efficacy. Evidence-based practice should not only include information and evidence regarding valid interventions and assessments but should also include the importance of intensity, frequency, and duration when establishing plans of care for pediatric clients. With healthcare evolving, new policies, and the Affordable Care Act [31], it is going to be increasingly important for OTs to provide guidelines of treatment frequency, intensity, and duration to ensure continuation of insurance coverage for occupational therapy services, in addition to demonstrating the effectiveness and value of occupational therapy services.

The findings of this study reflect the existing foundation of outcomes studies available regarding dosage and can foster reflection among occupational therapy practitioners. Such self-reflection may include guiding questions such as “what is the rationale for duration of the treatment, the number of sessions per week, and the number of minutes per session? Is what I have prescribed supported by the best possible evidence?”

Occupational therapy practitioners would benefit from paying closer attention to the parameters of dosage when exploring and implementing the evidence. They should consider the specific parameters of dosage (frequency, intensity, and duration), rationale for the dosage, and the setting of the intervention delivery within each article supporting their clinical practices.

### 4.8. Implications for Researcher

After reviewing 123 outcomes studies within occupational therapy specific journals there are several recommendations that researchers should consider as they disseminate findings related to occupational therapy based intervention outcomes applicable to pediatric populations or settings. Researchers should be explicit regarding the dosage parameters related to the intervention being assessed or independent variables measured. Further, it would be helpful if researchers include comments regarding the selection of dosage which may include how funding/reimbursement, time/resources, and/or setting may have been influencing factors. A clear description of how the study's methodology, dosage, and findings should or should not be interpreted and implemented in various practice settings is also needed. In addition to connecting the applicability of the findings to clinical practice, researchers should be explicit in the application of their study's methodology, dosage, and findings to better support replication.

### 4.9. Limitations

The limitations of this study include a heterogeneous sample, specifically diversity among intervention types, sample sizes, conditions/populations, and the journal's focus and specific aims. In addition, the literature search revealed some extreme outliers in all categories of the intervention dosage as noted in previous sections. Lastly, we are aware that the selection of the journals reviewed may result in a content bias since there may be relevant articles published in other sources that were not included within this study.

### 4.10. Recommendations for Future Research

Areas of future research should include analyzing the published research by adding categories related to setting (0–3 or early intervention, school-based, inpatient hospital and outpatient rehabilitation, and community based) and the type of research (case study or clinical). Analyzing unpublished theses and dissertations as they may include outcomes studies where the intervention did not result in significant findings and exploring their dosage may be advantageous to researchers and clinicians. Investigating outcomes studies back through 2002 may allow for the analysis to include changes in reporting of dosage and actual changes in dosage over time. Finally, evaluating pediatric outcomes studies that generated significant positive findings would be of value. In addition future research should include reviewing journals that are condition specific in an effort to establish the most effective interventions in addition to expanding the knowledge base in regard to the provision of occupational therapy services including dosage of such services. Categorizing and evaluating the funding source as a part of the outcome study (e.g., pro bono, public/private health insurance, government funded insurance, and grant funding) may also be beneficial. Including specific client factors such as severity of the physical, developmental or mental diagnosis, or occupational performance deficit would assist in the dosage discussion. Finally, the pediatric rehabilitation professions (occupational and physical therapy and speech language pathology) need to start exploring the possible links between dosage of an intervention and outcomes on an intervention by intervention and/or condition by condition basis.

## Figures and Tables

**Table 1:** Journal list.

Journal name	
American Journal of Occupational Therapy (AJOT)	
Canadian Journal of Occupational Therapy (CJOT)	
British Journal of Occupational Therapy (BJOT)	
Australian Occupational Therapy Journal (AOTJ)	
Occupational Therapy Journal of Research (OTJR)	
Physical and Occupational Therapy in Pediatrics (POTP)	
Occupational Therapy International (OTI)	
Journal of Occupational Therapy, Schools, & Early Intervention (JOTSEI)	
Scandinavian Journal of Occupational Therapy (SJOT)	

**Table 2:** Descriptive statistics.

Dosage subcomponents	Mean	Median	Standard deviation	Range
Sample size (*N* = 123) Number of participants	32.3	37.8	12	1–200

Session length (*n* = 108; 88%) Minutes	58.7	45	78.8	3–270

Duration of Plan of Care (*n* = 106; 86%) In weeks	12.1	10	12.5	0.14–96

Session frequency (*n* = 98; 80%) Number per week	3.4	2	3.3	1–10

Total hours of therapy (*n* = 92; 75%) Hours	18.1	10	20.3	0.34–100

**Table 3:** Levels of evidence.

Level of evidence	*n*
Level I: randomized control trials	14
Level II: cohort study, controlled trials not randomized	27
Level III: case-control study	26
Level IV: case-series, single-subject design	51
Level V: case report	6

**Table 4:** Articles reviewed.

Author(s)	Year	Article title	Journal
Bundy, Luckett, Naughton, Tranter, Wyver, Ragen, Singleton, and Spies	2008	“Playful Interaction: Occupational Therapy for All Children on the School Playground”	American Journal of Occupational Therapy

Cope, Forst, Bibis, and Liu	2008	“Modified Constraint-Induced Movement Therapy for a 12-Month-Old Child With Hemiplegia: A Case Report”	American Journal of Occupational Therapy

Drysdale, Casey, and Porter-Armstrong	2008	“Effectiveness of Training on the Community Skills of Children with Intellectual Disabilities”	Scandinavian Journal of Occupational Therapy

Lam, Wong, Fulks, and Holsti	2008	“Obsessional Slowness: A Case Study”	Canadian Journal of Occupational Therapy

Di Rezze, Wright, Curran, Campbell, and Macarthur	2008	“Individualized Outcome Measures for Evaluating Life Skill Groups for Children with Disabilities”	Canadian Journal of Occupational Therapy

Schultz-Kroh, Boener, Dinh, and Phelan	2008	“Handwriting Without Tears—A Short-Term Intervention for Children Living in a Homeless Shelter”	Journal of Occupational Therapy in Schools and Early Intervention

Lopez and Swinth	2008	“A Group Proprioceptive Program's Effect on Physical Aggression in Children”	Journal of Occupational Therapy in Schools and Early Intervention

Klein, Erickson, James, Perrott, Williamson, and Zacharuk	2008	“Effectiveness of a Computer Skills Program to Improve Written Communication in Children with Developmental Coordination Disorder”	Physical & Occupational Therapy in Pediatrics

Barnes, Vogel, Beck, Schoenfeld, and Owen	2008	“Self-Regulation Strategies of Children with Emotional Disturbance”	Physical & Occupational Therapy in Pediatrics

Eckman, Williams, Riegel, and Paul	2008	“Teaching Chewing: A Structured Approach”	American Journal of Occupational Therapy

Martin, Burtner, Poole, and Phillips	2008	“Case Report: ICF-Level Changes in a Preschooler After Constraint-Induced Movement Therapy”	American Journal of Occupational Therapy

Munguba, Valdes, and Bruno Da Silva	2008	“The Application of an Occupational Therapy Nutrition Education Programme for Children who are Obese”	Occupational Therapy International

Banks, Rodger, and Polatojko	2008	“Cognitive Orientation to (Daily) Occupational Performance: Changes in Strategy and Session Time Use Over the Course of Intervention”	Occupational Therapy Journal of Rehabilitation

Kang, Yoo, Chung, Jung, Chang, and Jeon	2008	“The Application of Client-Centered Occupational Therapy for Korean Children with Developmental Disabilities”	Occupational Therapy International

Pfeiffer, Henry, Miller, and Witherell	2008	“Effectiveness of Disc ‘O' Sit Cushions on Attention to Task in Second-Grade Students With Attention Difficulties”	American Journal of Occupational Therapy

Bazyk, Michaud, Goodman, Papp, Hawkins, and Welch	2009	“Integrating Occupational Therapy Services in a Kindergarten Curriculum: A Look at the Outcomes”	American Journal of Occupational Therapy

Nwora and Gee	2009	“A Case Study of a Five-Year-Old Child with Pervasive Developmental Disorder-Not-Otherwise Specified Using Sound-Based Interventions”	Occupational Therapy International

Qvarfordt, Engerstrom, and Eliasson	2009	“Guided Eating or Feeding: Three Girls with Rett Syndrome”	Scandinavian Journal of Occupational Therapy

Zwicker and Hadwin	2009	“Cognitive Versus Multisensory Approaches to Handwriting Intervention: A Randomized Controlled Trial”	Occupational Therapy Journal of Rehabilitation

Weintraub, Yinon, Hirsch, and Parush	2009	“Effectiveness of Sensorimotor and Task-Oriented Handwriting Intervention in Elementary School-Aged Students with Handwriting Difficulties”	Occupational Therapy Journal of Rehabilitation

Wuang, Wang, Huang, and Su	2009	“Prospective Study of the Effect of Sensory Integration, Neurodevelopmental Treatment, and Perceptual–Motor Therapy on the Sensorimotor Performance in Children With Mild Mental Retardation”	American Journal of Occupational Therapy

Roger, Pham, and Mitchell	2009	“Cognitive Strategy Use by Children with Asperger's Syndrome during Intervention for Motor-Based Goals”	Australian Occupational Therapy Journal

Rodger and Brandenburg	2009	“Cognitive Orientation to (Daily) Occupational Performance (CO-OP) with Children with Asperger's Syndrome Who Have Motor-Based Occupational Performance Goals”	Australian Occupational Therapy Journal

Lee, Muccio, and Osborne	2009	“The Effect of Chaining Techniques on Dressing Skills of Children with Moderate Mental Retardation: A Single-Subject Design Study”	Journal of Occupational Therapy in Schools and Early Intervention

Phelan, Steinke, and Mandich	2009	“Exploring a Cognitive Intervention for Children with Pervasive Developmental Disorder”	Canadian Journal of Occupational Therapy

Pizur-Barnekow, Kraemer, and Winters	2009	“Pilot Study Investigating Infant Vagal Reactivity and Visual Behavior During Object Perception”	American Journal of Occupational Therapy

Shimel, Candler, and Neville-Smith	2009	“Comparison of Cursive Handwriting Instruction Programs among Students without Identified Problems”	Physical & Occupational Therapy in Pediatrics

Silva, Ayres, Schalock, Bunse, and Budden	2009	“Qigong Massage Treatment for Sensory and Self-Regulation Problems in Young Children With Autism: A Randomized Controlled Trial”	American Journal of Occupational Therapy

Vaz, Daniela Virginia; Mancini, Marisa Cotta; do Amaral, Maira Ferreira; de Brito Brandao, Marina; de Franca Drummond, Adriana; and da Fonseca, Sergio Teixeira	2010	“Clinical Changes During an Intervention Based on Constraint-Induced Movement Therapy Principles on use of the Affected Arm of a Child with Obstetric Brachial Plexus Injury: A Case Report”	Occupational Therapy International

Gutman, Raphael, Ceder, Khan, Timp, and Salvant	2010	“The Effect of a Motor-Based, Social Skills Intervention for Adolescents with High-Functioning Autism: Two Single-Subject Design Cases”	Occupational Therapy International

Leew, Stein, and Gibbard	2010	“Weighted Vests' Effect on Social Attention for Toddlers with Autism Spectrum Disorders”	Canadian Journal of Occupational Therapy

Moir	2010	“Evaluating the Effectiveness of Different Environments on the Learning of Switching Skills in Children with Severe and Profound Multiple Disabilities”	British Journal of Occupational Therapy

Salem and Gropack	2010	“Aquatic Therapy for a Child with Type III Spinal Muscular Atrophy: A Case Report”	Physical & Occupational Therapy in Pediatrics

Missiuna, DeMatteo, Hanna, Mandich, Law, Mahoney, and Scott	2010	“Exploring the Use of Cognitive Intervention for Children with Acquired Brain Injury”	Physical & Occupational Therapy in Pediatrics

Shurtleff and Engsberg	2010	“Changes in Trunk and Head Stability in Children with Cerebral Palsy after Hippotherapy: A Pilot Study”	Physical & Occupational Therapy in Pediatrics

MaKay, McCluskey, and Mayes	2010	“The Log Handwriting Program in Improved Children's Writing Legibility”	American Journal of Occupational Therapy

Watson, Ito, Smith, and Andersen	2010	“Effect of Assistive Technology in a Public School Setting”	American Journal of Occupational Therapy

Costigan and Light	2010	“Effect of Seated Position on Upper Extremity Access to Augmentative Communication for Children with CP”	American Journal of Occupational Therapy

Roberts, Siever, and Mair	2010	“Effects of Kinesthetic Cursive Handwriting Intervention for Grade 4–6 Students”	American Journal of Occupational Therapy

Ryan, Rigby, and Campbell	2010	“Randomized Controlled Trial Comparing Two School Furniture Configurations in the Printing Performance of Young Children with Cerebral Palsy”	Australian Occupational Therapy Journal

Hwang, Lin, Coster, Bigsby, and Vergara	2010	“Effectiveness of Check and Jaw Support to Improve Feeding Performance of Preterm Infants”	American Journal of Occupational Therapy

Bagatell, Mirigliani, Patterson, Reyes, and Test	2010	“Effectiveness of Therapy Ball Chairs on Classroom Participation in Children with ASD”	American Journal of Occupational Therapy

Chard and Pierse	2011	“The Effect of Introducing Non-Play Items into a Primary School Playground in Ireland”	Journal of Occupational Therapy in Schools and Early Intervention

Wilkes, Cordier, Bundy, Docking, and Munro	2011	“A Play-based Intervention for Children with ADHD: A Pilot Study”	Australian Occupational Therapy Journal

Meyer, Rice, and Metz	2011	“Knowledge of Results and Learning to Tell Time with Typically Developing 7- and 8-Year-Old Children: A Single Subject Research Design”	Journal of Occupational Therapy in Schools and Early Intervention

Benson, Beeman, Smitsky, and Provident	2011	“The Deep Pressure and Proprioceptive Technique (DPPT) versus Nonspecific Child-Guided Brushing: A Case Study”	Journal of Occupational Therapy in Schools and Early Intervention

Dunford	2011	“Goal-Oriented Group Intervention for Children with Developmental Coordination Disorder”	Physical & Occupational Therapy in Pediatrics

Dionne and Martini	2011	“Floor Time Play with a Child with Autism: A Single-Subject Study”	Canadian Journal of Occupational Therapy

Bates	2011	“Congenital Diaphragmatic Hernia and Occupational Therapy: A Case Report”	Physical & Occupational Therapy in Pediatrics

Pfeiffer, Koeing, Kinnealey, Sheppard, and Henderson	2011	“Effectiveness of Sensory Integration Interventions in Children with Autism Spectrum Disorders: A Pilot Study”	American Journal of Occupational Therapy

Rowe, Candler, and Neville	2011	“Noise Reduction Headphones and Autism: A Single Case Study”	Journal of Occupational Therapy in Schools and Early Intervention

Taras, Brennan, Gilbert, and Reed	2011	“Effectiveness of Occupational Therapy Strategies for Teaching Handwriting Skills to Kindergarten Children”	Journal of Occupational Therapy in Schools and Early Intervention

Umeda and Deitz	2011	“Effects of Therapy Cushions on Classroom Behaviors of Children with Autism Spectrum Disorder”	American Journal of Occupational Therapy

Hahn-Markowitz, Manor, and Maeir	2011	“Effectiveness of Cognitive–Functional (Cog–Fun) Intervention with Children With Attention Deficit Hyperactivity Disorder: A Pilot Study”	American Journal of Occupational Therapy

Fedewa and Erwin	2011	“Stability Balls and Students with Attention and Hyperactivity Concerns: Implications for On Task and In Seat behaviors”	American Journal of Occupational Therapy

Golos, Sarid, Weill, and Weintraub	2011	“Efficacy of an Early Intervention Program for At Risk Preschool Boys: A Two-Group Control Study”	American Journal of Occupational Therapy

Lust and Donica	2011	“Effectiveness of a Handwriting Readiness Program in Head Start: A Two-Group Controlled Trial”	American Journal of Occupational Therapy

Silva, Schalock, and Gabrielson	2011	“Early Intervention for Autism with a Parent-Delivered Qigong Massage Program: A Randomized Controlled Trial”	American Journal of Occupational Therapy

Stagnitti O'Connor and Sheppard	2012	“Impact of the Learn to Play Program on Play, Social Competence, and Language for Children Aged 5–8 Years Who Attend a Specialist School”	Australian Occupational Therapy Journal

Sawatzky, Rushton, Denison, and McDonald	2012	“Wheelchair Skills Training Programme for Children: A Pilot Study”	Australian Occupational Therapy Journal

Shurtleff and Engsberg	2012	“Long-Term Effects of Hippotherapy on One Child with Cerebral Palsy: A Research Case Study”	British Journal of Occupational Therapy

Schneck, Shasby, Meyers, and DePoy	2012	“Handwriting without Tears versus Teacher-Designed Handwriting Instruction in First Grade Classrooms”	Journal of Occupational Therapy, Schools, & Early Intervention

Lawson, Cox, and Blackwell	2012	“Yoga as a Classroom Intervention for Preschoolers”	Journal of Occupational Therapy, Schools, & Early Intervention

Reidy, Naber, Viguers, Allison, Brady, Carney, Salorio, and Pidock	2012	“Outcomes of a Clinic-Based Pediatric Constraint-Induced Movement Therapy Program”	Physical & Occupational Therapy in Pediatrics

Aarts, Pauline B.; Hartingsveldt, Margo; Anderson, Patricia G.; Tillaar, Ingrid; Burg, Jan; and Geurts, Alexander	2012	“The Pirate Group Intervention Protocol: Description and a Case Report of a Modified Constraint-Induced Movement Therapy Combined with Bimanual Training for Young Children with Unilateral Spastic Cerebral Palsy”	Occupational Therapy International

Brandão, Gordon, & Mancini	2012	“Functional Impact of Constraint Therapy and Bimanual Training in Children with Cerebral Palsy: A Randomized Controlled Trial”	American Journal of Occupational Therapy

Palsbo and Hood-Szivek	2012	“Effect of Robotic-Assisted Three-Dimensional Repetitive Motion to Improve Hand Motor Function and Control in Children with Handwriting Deficits: A Nonrandomized Phase 2 Device Trial”	American Journal of Occupational Therapy

Case-Smith, Holland, Lane, and White	2012	“Effect of a Coaching Handwriting Program for First Graders: One-Group Pretest–Posttest Design”	American Journal of Occupational Therapy

Dunn, Cox, Foster, Mische-Lawson, and Tanquary	2012	“Impact of a Contextual Intervention on Child Participation and Parent Competence among Children with Autism Spectrum Disorders: A Pretest–Posttest Repeated-Measures Design”	American Journal of Occupational Therapy

Gutman, Raphael, and Rao	2012	“Effect of a Motor Based Role Play Intervention on the Social Behaviors of Adolescents with High Functioning ASD: Multiple Baseline Single Subject Design”	American Journal of Occupational Therapy

Koenig, Buckley-Reen, and Garg	2012	“Efficacy of the Get Ready to Learn Yoga Program among Children with ASD: A Pretest-Posttest Control Group Design”	American Journal of Occupational Therapy

Schaaf, Hunt, and Benevides	2012	“Occupational Therapy Using SI to Improve Participation of a Child with ASD: A Case Report”	American Journal of Occupational Therapy

Kinnealey, Pfeiffer, Miller, Roan, Shoener, and Ellner	2012	“Effect of Classroom Modification on Attention and Engagement of Students with Autism or Dyspraxia”	American Journal of Occupational Therapy

Silva, Schalock, Garberg, and Lammers-Smith	2012	“Qigong Massage for Motor Skills in Young Children with Cerebral Palsy and Down Syndrome”	American Journal of Occupational Therapy

Ajzenman, Standeven, and Shurtleff	2013	“Effect of Hippotherapy on Motor Control, Adaptive Behaviors, and Participation in Children with Autism Spectrum Disorder: A Pilot Study”	American Journal of Occupational Therapy

Howe, Roston, Sheu, and Hinojosa	2013	“Assessing Handwriting Intervention Effectiveness in Elementary School Students: A Two-Group Controlled Study”	American Journal of Occupational Therapy

Bellows, Davies, Anderson, and Kennedy	2013	“Effectiveness of a Physical Activity Intervention for Head Start Preschoolers: A Randomized Intervention Study”	American Journal of Occupational Therapy

Rowe, Yuen, and Dure	2013	“Comprehensive Behavioral Intervention to Improve Occupational Performance in Children with Tourette Disorder”	American Journal of Occupational Therapy

Wu, Hung, Tseng, and Huang	2013	“Group Constraint-Induced Movement Therapy for Children with Hemiplegic Cerebral Palsy: A Pilot Study”	American Journal of Occupational Therapy

Tsai, Meng, Wu, Jang, and Su	2013	“Effects of Visual Rehabilitation on a Child with Severe Visual Impairment”	American Journal of Occupational Therapy

Ohl, Graze, Weber, Kenny, Salvatore, and Wagreich	2013	“Effectiveness of a 10-Week Tier-1 Response to Intervention Program in Improving Fine Motor and Visual-Motor Skills in General Education Kindergarten Students”	American Journal of Occupational Therapy

Tokolahi, Em-Chhour, Barkwill, and Stanley	2013	“An Occupation-Based Group for Children with Anxiety”	British Journal of Occupational Therapy

Monahan, Classen, and Helsel	2013	“Pre-driving Evaluation of a Teen with Attention Deficit Hyperactivity Disorder and Autism Spectrum Disorder”	Canadian Journal of Occupational Therapy

Olsen, Ross, Foreman, and Engsberg	2013	“Changes in Muscle Activation following Ankle Strength Training in Children with Spastic Cerebral Palsy: An Electromyography Feasibility Case Report”	Physical and Occupational Therapy in Pediatrics

Qualls, Arnold, McEwen, and Jefferies	2013	“Exercise Using the Wii Fit Plus with a Child with Primary Raynaud's Disease and Obesity: A Case Report”	Physical and Occupational Therapy in Pediatrics

Thompson and Johnston	2013	“Use of Social Stories to Improve Self-Regulation in Children with Autism Spectrum Disorders”	Physical and Occupational Therapy in Pediatrics

Kurz, Stuberg, DeJong, and Arpin	2013	“Overground Body-Weight-Supported Gait Training for Children and Youth with Neuromuscular Impairments”	Physical and Occupational Therapy in Pediatrics

Potasz, Varela De Varela, Coin De Carvalho, Fernandes Do Prado, and Fernades Do Prado	2013	“Effect of Play Activities on Hospitalized Children's Stress: A Randomized Clinical Trial”	Scandinavian Journal of Occupational Therapy

Lee, Grey, Ora, Stren, and Sytner	2013	“The Effect of Computer-Based Intervention on Enhancing Visual Perception of Preschool Children with Autism: A Single-Subject Design Study”	Journal of Occupational Therapy in Schools and Early Intervention

Kaiser	2013	“Children with Developmental Coordination Disorder: The Effects of Combined Intervention on Motor Coordination, Occupational Performance, and Quality of Life”	Journal of Occupational Therapy in Schools and Early Intervention

Donica, Golns, and Wagner	2013	“Effectiveness of Handwriting Readiness Programs on Postural Control, Hand Control, and Letter and Number Formation in Head Start Classrooms”	Journal of Occupational Therapy in Schools and Early Intervention

Sheppard, Osmond, and Stagnitti	2013	“The Effectiveness of a Multidisciplinary Intervention to Improve School Readiness in Children with Developmental Concerns: Children's Skill Development and Parent Perspective”	Journal of Occupational Therapy in Schools and Early Intervention

Bhopti and Brown	2013	“Examining the Wilbargers' Deep Pressure and Proprioceptive Technique for Treating Children with Sensory Defensiveness Using a Multiple-Single-Case Study”	Journal of Occupational Therapy in Schools and Early Intervention

Garg, Buckley-Reen, Alexander, Chintakrindi, Venice, and Koeng	2013	“The Effectiveness of Manualized Yoga Intervention on Classroom Behaviors in Elementary School Children with Disabilities: A Pilot Study”	Journal of Occupational Therapy in Schools and Early Intervention

Delegato, McLaughline, Derby, and Schuster	2013	“The Effects of Using Handwriting without Tears and a Handwriting Racetrack to Teach Five Preschool Students with Disabilities Pre Handwriting and Handwriting”	Journal of Occupational Therapy in Schools and Early Intervention

Kennedy-Behr, Rodger, Graham, and Mickan	2013	“Creating Enabling Environments at Preschool for Children with Developmental Coordination Disorder”	Journal of Occupational Therapy in Schools and Early Intervention

Hilton, Cumpata, Klohr, Gaetke, Artner, Johnson, and Dobbs	2014	“Effects of Exergaming on Executive Function and Motor Skills in Children with Autism Spectrum Disorder: A Pilot Study”	American Journal of Occupational Therapy

Wilkes-Gilen, Bundy, Cordier, and Lincoln	2014	“Evaluation of a Pilot Parent-Delivered Play-Based Intervention for Children with Attention Deficit Hyperactivity Disorder”	American Journal of Occupational Therapy

Smith, Weaver, and Holland	2014	“Effects of a Classroom-Embedded Occupational Therapist–Teacher Handwriting Program for First-Grade Students”	American Journal of Occupational Therapy

Orban, Erlandsson, Edberg, Onnerfalt, and Thorngren-Jerneck	2014	“Effect of an Occupation-Focused Family Intervention on Change in Parents' Time Use and Children's Body Mass Index”	American Journal of Occupational Therapy

Maeir, Fisher, Bar-Llan, Boas, Berger, and Landau	2014	“Effectiveness of Cognitive–Functional (Cog–Fun) Occupational Therapy Intervention for Young Children with Attention Deficit Hyperactivity Disorder: A Controlled Study”	American Journal of Occupational Therapy

Lin, Lee, Chang, and Hong	2014	“Effects of Weighted Vests on Attention, Impulse Control, and On-Task Behavior in Children with Attention Deficit Hyperactivity Disorder”	American Journal of Occupational Therapy

Lane, Ivey, and May-Bensen	2014	“Test of Ideational Praxis (TIP): Preliminary Findings and Interrater and Test–Retest Reliability with Preschoolers”	American Journal of Occupational Therapy

Roberts, Derkach-Ferguson, Siever, and Rose	2014	“An Examination of the Effectiveness of Handwriting Without Tears® Instruction”	Canadian Journal of Occupational Therapy

Case-Smith, Holland, and White	2014	“Effectiveness of a Co-Taught Handwriting Program for First Grade Students”	Physical and Occupational Therapy in Pediatrics

Pollock, Sharma, Christenson, Law, Gorter, and Darrah	2014	“Change in Parent-Identified Goals in Young Children with Cerebral Palsy Receiving a Context-Focused Intervention: Associations with Child, Goal and Intervention Factors”	Physical and Occupational Therapy in Pediatrics

McConnel, Johnston, and Kerr	2014	“Efficacy and Acceptability of Reduced Intensity Constraint-Induced Movement Therapy for Children Aged 9–11 Years with Hemiplegic Cerebral Palsy: A Pilot Study”	Physical and Occupational Therapy in Pediatrics

Lowes, Mayhan, Orr, Batterson, Tonneman, Meyer, and Case-Smith	2014	“Pilot Study of the Efficacy of Constraint-Induced Movement Therapy for Infants and Toddlers with Cerebral Palsy”	Physical and Occupational Therapy in Pediatrics

Clark and Luze	2014	“Predicting Handwriting Performance in Kindergarteners Using Reading, Fine-Motor, and Visual-Motor Measures”	Journal of Occupational Therapy in Schools and Early Intervention

Cobb, Fitzgerald, Lanigan-O-Keeffe, Irwin, and Mellerick	2014	“Students with Social, Emotional, and Behavioral Difficulties: The Alert Program Trial in Post-Primary Schools”	Journal of Occupational Therapy in Schools and Early Intervention

Iwanaga, Honda, Nakane, Tanaka, Toeda, and Tanaka	2014	“Pilot Study: Efficacy of Sensory Integration Therapy for Japanese Children with High-Functioning Autism Spectrum Disorder”	Occupational Therapy International

Gee, Thompson, and St. John	2014	“Efficacy of a Sound-Based Intervention with a Child with an Autism Spectrum Disorder and Auditory Sensory Over-Responsivity”	Occupational Therapy International

An	2014	“Occupation-Based Family-Centered Therapy Approach for Young Children with Feeding Problems in South Korea; A Case Study”	Occupational Therapy International

Janeslatt, Kottorp, and Granlund	2014	“Evaluating Intervention Using Time Aids in Children with Disabilities”	Scandinavian Journal of Occupational Therapy

Almomani, Josman, Al-Momani, Malkawi, Nazzal, Almahdawi, and Almomani	2014	“Factors Related to Cognitive Function among Elementary School Children”	Scandinavian Journal of Occupational Therapy

Liao, Hwang, Chen, Lee, Chen, and Lin	2014	“Home-Based DIR/Floortime*™* Intervention Program for Preschool Children with Autism Spectrum Disorders: Preliminary Findings”	Physical and Occupational Therapy in Pediatrics

Hape, Flood, McArthur, Sidara, Stephens, and Welsch	2014	“A Pilot Study of the Effectiveness of the Handwriting without Tears® Curriculum in First Grade”	Journal of Occupational Therapy in Schools and Early Intervention

Blackwell, Yeager, Lawson, Bird, and Cook	2014	“Teaching Children Self-Regulation Skills within the Early Childhood Education Environment: A Feasibility Study”	Journal of Occupational Therapy in Schools and Early Intervention

Candler, Mulder, and Nall	2014	“Embedding Video-Based Modeling Handwriting Instruction in a Montessori Preschool Phonics Program”	Journal of Occupational Therapy in Schools and Early Intervention

Stewart and Umeda	2014	“Video Modeling in Occupational Therapy for Very Young Children with Autism Spectrum Disorder: A Pilot Study”	Journal of Occupational Therapy in Schools and Early Intervention

Chen, Lin, Kang, Wu, Chen, and Hsieh	2014	“Potential Predictors of Functional Outcomes after Home-Based Constraint-Induced Therapy for Children with Cerebral Palsy”	American Journal of Occupational Therapy

## Appendix

See Table 4.

## References

[1] American Occupational Therapy Association (2014). Occupational therapy practice framework: domain and process (3rd edition). *The American Journal of Occupational Therapy*.

[2] Kolobe T. H. A., Christy J. B., Gannotti M. E. (2014). Research summit III proceedings on dosing in children with an injured brain or cerebral palsy: executive summary. *Physical Therapy*.

[3] Gannotti M. E., Christy J. B., Heathcock J. C., Kolobe T. H. A. (2014). A path model for evaluating dosing parameters for children with cerebral palsy. *Physical Therapy*.

[4] Pescatello L. S., Arena R., Riebe D., Thompson P. D. (2014). *ACSM's Guidelines for Exercise Testing and Prescription*.

[5] Hayward K. S., Barker R. N., Wiseman A. H., Brauer S. G. (2013). Dose and content of training provided to stroke survivors with severe upper limb disability undertaking inpatient rehabilitation: an observational study. *Brain Impairment*.

[6] Waddell K. J., Birkenmeier R. L., Moore J. L., Hornby T. G., Lang C. E. (2014). Feasibility of high-repetition, task-specific training for individuals with upper-extremity paresis. *American Journal of Occupational Therapy*.

[7] May-Benson T. A., Koomar J. A. (2010). Systematic review of the research evidence examining the effectiveness of interventions using a sensory integrative approach for children. *The American Journal of Occupational Therapy*.

[8] Kirk-Sanchez N. J., Roach K. E. (2001). Relationship between duration of therapy services in a comprehensive rehabilitation program and mobility at discharge in patients with orthopedic problems. *Physical Therapy*.

[9] Case-Smith J., O'Brien J. (2010). *Occupational Therapy for Children*.

[10] Lane S., Bundy A. (2012). *Kids Can be Kids: A Childhood Occupations Approach*.

[11] Hart D. L., Tepper S., Lieberman D. (2001). Changes in health status for persons with wrist or hand impairments receiving occupational therapy or physical therapy. *American Journal of Occupational Therapy*.

[12] Kwakkel G. (2006). Impact of intensity of practice after stroke: issues for consideration. *Disability and Rehabilitation*.

[13] Latham N. K., Jette D. U., Coster W. (2006). Occupational therapy activities and intervention techniques for clients with stroke in six rehabilitation hospitals. *The American Journal of Occupational Therapy*.

[14] Palisano R. J., Murr S. (2009). Intensity of therapy services: what are the considerations?. *Physical and Occupational Therapy in Pediatrics*.

[15] Feder K. P., Racine M. B., Majnemer A. (2008). A review of handwriting performance and interventions: does remediation work. *Israeli Journal of Occupational Therapy*.

[16] Warren S. F., Fey M. E., Yoder P. J. (2007). Differential treatment intensity research: a missing link to creating optimally effective communication interventions. *Mental Retardation and Developmental Disabilities Research Reviews*.

[17] Kaminker M. K., Chiarello L. A., O'Neil M. E., Dichter C. G. (2004). Decision making for physical therapy service delivery in schools: aa nationwide survey of pediatric physical therapists. *Physical Therapy*.

[18] Bates A. C. (2011). Congenital diaphragmatic hernia and occupational therapy: a case report. *Physical and Occupational Therapy in Pediatrics*.

[19] Ryan S. E., Rigby P. J., Campbell K. A. (2010). Randomised controlled trial comparing two school furniture configurations in the printing performance of young children with cerebral palsy. *Australian Occupational Therapy Journal*.

[20] Pizur-Barnekow K., Kraemer G. W., Winters J. M. (2008). Pilot study investigating infant vagal reactivity and visual behavior during object perception. *The American Journal of Occupational Therapy*.

[21] de Brito Brandão M., Gordon A. M., Mancini M. C. (2012). Functional impact of constraint therapy and bimanual training in children with cerebral palsy: a randomized controlled trial. *The American Journal of Occupational Therapy*.

[22] Classen S., Monahan M., Wang Y. (2013). Driving characteristics of teens with attention deficit hyperactivity and autism spectrum disorder. *American Journal of Occupational Therapy*.

[23] Nwora A. J., Gee B. M. (2009). A case study of a five-year-old child with pervasive developmental disorder-not otherwise specified using sound-based interventions. *Occupational Therapy International*.

[24] Portney L. G., Watkins M. P. (2008). *Foundations of Clinical Research: Applications to Practice*.

[25] Novak I. (2012). Evidence to practice commentary: is more therapy better?. *Physical and Occupational Therapy in Pediatrics*.

[26] Palisano R. J., Murr S. (2009). Intensity of therapy services: what are the onsiderations. *Physical and Occupational Therapy in Pediatrics*.

[27] World Health Organization (2002). *The World Health Report 2002: Reducing Risks, Promoting Healthy Life*.

[28] Bailes A. F., Reder R., Burch C. (2008). Development of guidelines for determining frequency of therapy services in a pediatric medical setting. *Pediatric Physical Therapy*.

[29] Clark F. (2010). High-definition occupational therapy: HD OT. *The American Journal of Occupational Therapy*.

[30] American Occupational Therapy Association (2015). Occupational therapy code of ethics (2015). *American Journal of Occupational Therapy*.

[31] Patient Protection and Affordable Care Act

